# Supplementary material for: Identification of photoperiod-induced specific miRNAs in the adrenal glands of Sunite sheep (Ovis aries)
Source: Front Vet Sci. 2022 Jul 22;9:888207. doi: 10.3389/fvets.2022.888207 (PMC9354845; doi:10.3389/fvets.2022.888207)
Supplement: Supplementary file 1 [file Data_Sheet_1.ZIP › Supplementary file/miRDeep2 file.zip!/miRDeep2 file/Know_Structure/LP42A3/oar-mir-668.pdf]

miRBase precursor : oar-mir-668
Total read count : 37
oar-miR-668-5p read count : 4
oar-miR-668-3p read count : 33
remaining reads : 0

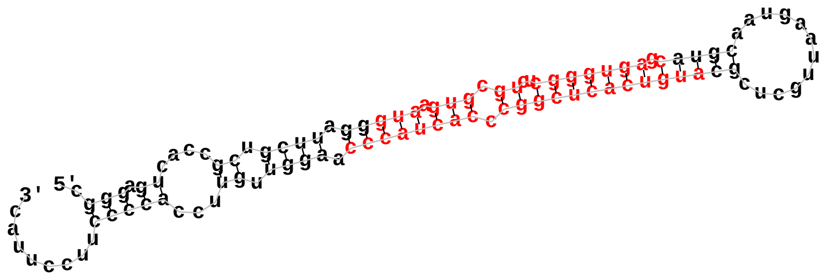

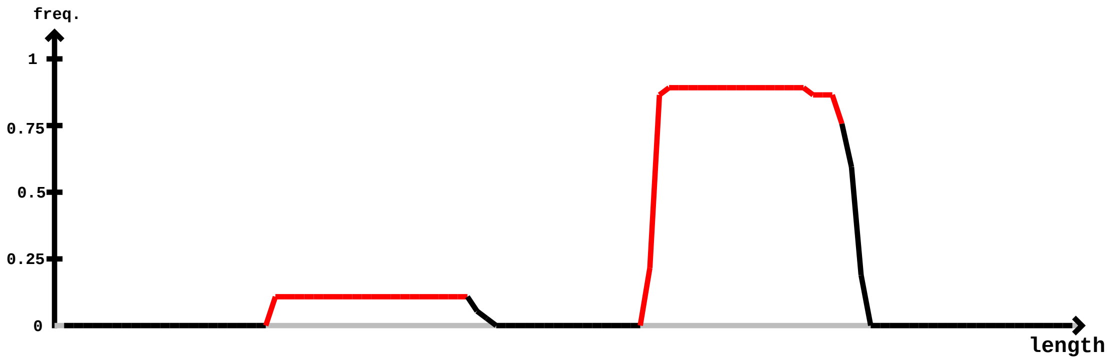


```
                                                            oar-miR-668-3p
                     oar-miR-668-5p
5'- cgggagucaccgcugcuuaggguaagugcgucucgggugagcaugcaaugaauugcucgcaugucacucggcccacuacccaagguuguuccaccccuuccuuac   -3'    exp
    .(((.((....((.((((.(((((.(((.((..(((((((.(((((............)))))))))))))).)))))))).)))).))...)))))........    reads     mm        sample
    .....................guaagugcgucucgggugagc...............................................................    2         0         seq
    .....................guaagugcgucucgggugagca..............................................................    1         0         seq
    .....................guaagugcgucucgggugagcau.............................................................    1         0         seq
    ............................................................augucacucggcccacu............................    1         0         seq
    ............................................................augucacucggcccacuaccc........................    2         0         seq
    ............................................................augucacucggcccacuaccca.......................    4         0         seq
    ............................................................augucacucggcccacuacccaa......................    1         0         seq
    .............................................................ugucacucggcccacuacc.........................    4         0         seq
    .............................................................ugucacucggcccacuaccc........................    4         0         seq
    .............................................................ugucacucggcccacuaccca.......................    11        0         seq
    .............................................................ugucacucggcccacuacccaa......................    5         0         seq
    ..............................................................gucacucggcccacuacccaa......................    1         0         seq
```
